# Radiation exposure in emergency ureteric stenting: A subgroup analysis by operator

**DOI:** 10.1002/bco2.245

**Published:** 2023-04-29

**Authors:** Kevin Tree, Nicholas Chang, Roy Huynh, Balasubramaniam Indrajit, Dean Fisher, Sris Baskaranathan

**Affiliations:** ^1^ Department of Surgery Dubbo Base Hospital Dubbo Australia; ^2^ University of Newcastle Newcastle Australia; ^3^ University of Sydney Sydney Australia

**Keywords:** radiation dosage, stents, ureteric stones, urolithiasis, urology

## Abstract

**Objectives:**

To review radiation exposure during emergency ureteric stent insertion to identify differences based on operator experience, specialty operator and stone characteristics.

**Patients and methods:**

A retrospective audit over 10 years was performed for patients who underwent emergency stent insertion for urolithiasis with intraoperative fluoroscopy. Outcomes measured included operator experience, radiation exposure (mGy), dose area product (Gy/cm^2^), fluoroscopy time, stone characteristics and patient BMI. Analysis was performed in IBM SPSS Version 28. *p* < 0.05 was considered statistically significant.

**Results:**

Four hundred ten patients were identified, with a median age of 57 years, 64.6% male and a median BMI of 30. Urolithiasis was left‐sided in 50.8%, with a median size of 7 mm and predominantly proximal (49%) followed by mid (34.5%) and distal (12.1%) location. Median radiation exposure was 12.6 mGy, 2.94 Gy/cm^2^ and fluoroscopy time 44.5 s, with no significant difference between consultants and registrars. No significant association between radiation exposure for subgroups of stone location, gender, size, laterality or specialty registrar (general surgery vs. urology).

**Conclusion:**

No significant difference in radiation exposure was identified between registrars and consultants or between subspecialty registrars. We suggest formal radiation safety education for all health professionals involved with intra‐operative fluoroscopy and personal dosimeters.

## 1 INTRODUCTION

Ureteric stenting is performed to relieve upper urinary tract obstruction and most commonly for urolithiasis. International guidelines^1^, ^2^, ^3^ recommend urgent emergency ureteric stent insertion to relieve obstruction in sepsis and anuria. Using stone size as a predictor, 95% of stones up to 4 mm pass within 40 days^3^ but the likelihood of spontaneous stone expulsion decreases with increased stone size, with only 62% of stones >5 mm passing spontaneously despite medical expulsion therapy. Relative indications for interventions include ongoing pain, renal impairment and stone size not amenable to spontaneous passage. In Australia 2020–2021, 34 690 ureteric stents were inserted in Australia^4^ and 61 300 diagnosed cases of urolithiasis required hospital attendance.^5^ The burden of the disease urolithiasis has increased in Australia over the years^6^ with a corresponding increase in endoscopic procedures and fluoroscopy.

The Australian Radiation Protection and Nuclear Safety Agency (ARPANSA) have a 20 mSv dose limit for occupationally exposed workers^7^ in the medical field. To minimise exposure and mitigate risk for patients and workers the ‘as low as reasonably achievable’ (ALARA) principles are applied through practises such as minimising fluoroscopy time (FT), maximising distance from equipment and use of shielding. The literature demonstrates that lead apron and thyroid shield provides a 97%–98% dose reduction during retrograde endourological procedures.^8^ Ureteric stenting, a common endourological procedure, requires intra‐operative fluoroscopy to demonstrate anatomy, identify obstruction and ensure appropriate stent placement. A frequent and usually simple procedure, surgical registrars often learn to perform this operation independently throughout their training under the supervision of urologists. However, within the Australian surgical training curriculum, radiation safety courses are only mandatory for orthopaedics and vascular surgery registrars, but not for urology and general surgery. Instead, radiation safety is supervised by hospital‐based Ionising Radiation Safety Committees, which can support registrars to gain appropriate education and accreditation. Internationally, audits have been conducted to develop reference standards^9^ for radiation exposure in urological procedures to complement ALARA principles and mitigate radiation risk to patients and operators.

This study aims to determine radiation exposure during emergency ureteric stent insertion based on surgeon experience and specialty. We hypothesise that for emergency ureteric stenting, radiation exposure would be increased for less experienced operators such as registrars and non‐urology primary operators. Furthermore, we examine other risk factors for increased radiation exposure and assess intraoperative radiation exposure in reference to the FLASH study^9^ reference levels for ureteric stent insertion.

## 2 METHODS

A retrospective audit was performed from February 2012 to December 2022 at Dubbo Base Hospital, a large rural referral centre and teaching hospital in New South Wales, Australia. From February 2020 to December 2022, an unaccredited urology registrar role was introduced, with the role's responsibilities previously performed by an accredited general surgery registrar (Data S1). Registrars underwent 6 to 12 month placements, and all urology procedures were performed under the supervision of a urologist.

Patients were identified using Cerner's application electronic medical records for all theatre events coded as emergency ureteric stent insertions. Baseline demographic information, primary operator, case duration, stone size and location, radiation exposure, case duration and operative details were recorded. Indications for ureteric stent insertion followed international guideline recommendations EAU, AUA and BAUS.

Exclusion criteria were patients who presented with obstruction not due to urolithiasis, bilateral disease, or if primary removal of the stone was performed. Cases were excluded if the stent was elective or not the primary operation. Pregnant patients and paediatric patients less than 18 years old were excluded as high‐risk patients.

The research protocol was approved by the Greater Western Human Ethics Research Committee as a negligible/low‐risk project. Data was de‐identified and retrospectively collected, and therefore informed consent was not required from each patient. Research Ethics and Governance Information System (REGIS) Project identifier ETH02586, PID02907 and STE04126.

### 2.1 Imaging and radiation

Intraoperative imaging was performed by a radiographer under the instruction of the operator using an image intensifier. The image intensifier used was a Philips BV Pulsera mobile C‐arm throughout the study time period and a second mobile C‐arm Philips Zenition 70 in 2022. Both had a Monoblock 80 Khz high frequency generator with a maximum output of 15 kW. Fluoroscopy data was collected from the electronic medical imaging records DICOM Radiation Dose Structured Report (RDSR) generated by the C‐arm. Radiation exposure was defined in the outcomes mGy, Gy/cm^2^ dose area product (DAP) and FT as per FLASH study^9^ reference outcomes. All members of the operating team in an operation wear RADsafe non‐lead gowns and thyroid collars with 0.50 mm equivalent lead protection. These are compliant with international radiation safety standards and are tested annually to ensure compliance.

Stone size was recorded based on the radiologist's report with the maximum dimension chosen. When multiple stones are present, the obstructing stone was selected. If ambiguity of stone size or location was identified, a consensus decision was made with two data coders and an external reviewer. Location within the ureter was divided into proximal, middle and distal using the anatomical landmarks of the sacrum's upper and lower border to divide the three segments.

### 2.2 Primary operator

The primary operator was determined from documentation of a ‘primary proceduralist’ on operation reports from Cerner's electronic medical records programme SurgiNet. Where a registrar was documented as primary and the consultant urologist was documented as the ‘supervising proceduralist’ the registrar was considered the primary operator. If two primary operators were documented, the more senior operator was selected as the primary.

### 2.3 Primary and secondary outcomes

The primary outcome was to determine whether a difference existed in radiation exposure (mGy, DAP and FT) and fluoroscopy usage for subgroups of operator seniority, speciality, BMI, stone size and location, or case duration.

The secondary outcomes measured were to compare mGy, DAP and FT between the time periods of 2012–2019 and 2020–2022 with urology and general surgery registrars, respectively. Additionally, a risk factor analysis for stone size was performed.

### 2.4 Statistical analysis

SPSS version 28^10^ and Microsoft Excel were used for statistical analysis. The Shapiro–Wilk test was performed along with a data histogram to determine normality. Independent *t*‐test and chi‐squared tests were performed to compare baseline characteristics with normal distribution, and non‐parametric Mann–Whitney test if not. Bivariate Pearson correlation was performed to compare outcomes of subgroups for continuous data. For outcomes with greater than 2 variables, a comparison was performed with one‐way ANOVA testing. The pairwise deletion was performed to account for missing data. A *p*‐value of <0.05 was considered statistically significant. Correlation strength <0.2 was considered very low, 0.2–0.4 low, 0.4–0.6 moderate, 0.6–0.8 strong and 0.8–1.0 very strong.

## 3 RESULTS

Six hundred sixty‐five patients were identified with 255 patients meeting the exclusion criteria. The most common reasons for exclusion were non‐urolithiasis obstruction, incomplete data or primary ureteroscopy performed. Four hundred ten patients were included, median age of 57 years (interquartile range [IQR] 23), 64.6% male and a median BMI of 30 (IQR 8.6). Urolithiasis was left‐sided in 50.8%, with a median size of 7 mm (IQR 4 mm) and stone location predominantly proximal (49%) followed by mid (34.5%) and distal (12.1%). Radiation exposure was a median of 12.6 mGy (IQR 13.9 mGy), a median DAP of 2.94 Gy/cm^2^ (IQR 3.48 Gy/cm^2^) and FT median of 44.5 s (IQR 44 s).

### 3.1 Primary operator

Two hundred forty‐eight operations were performed by three consultants compared to 162 by 17 registrars. No significant difference in age, gender, laterality or stone size was identified (Table 1). Patient BMI was higher in consultant operations (*p* = 0.047). Registrar operations trended to having procedures with a greater proportion of distal ureter stones but this was not significant (*p* = 0.057). No significant difference in mGy (*p* = 0.671), DAP (*p* = 0.613) or FT (*p* = 0.266) was identified (Table 3).

**TABLE 1:** Baseline characteristics comparison between consultant and registrar.

	Consultant	Registrar	*p*‐value
Patients	248	162	
Age, years, median (IQR)	57 (21)	56 (27)	0.269
Gender, male (%)	173 (69.8)	104 (64.2)	0.240
BMI, median (IQR)	30.6 (8)	29.4 (8.7)	0.047
Left side, *n* (%)	138 (55.6)	80 (49.4)	0.215
Case duration, minutes, median (IQR)	41 (14)	43 (15)	0.027
Size of stone, mm, median (IQR)	7 (4)	7 (5)	0.829
Stone location			0.057
Proximal, *n* (%)	133 (53.6)	77 (47.5)	
Mid, *n* (%)	93 (37.5)	55 (34)	
Distal, *n* (%)	22 (8.9)	30 (18.5)	

Abbreviation: IQR, interquartile range.

### 3.2 Specialty registrar

One hundred twenty‐six patients were identified in the 2020–2022 period compared with 284 patients for 2012–2019 (Table 2). Patients in the contemporary time period were significantly older (*p* = 0.001) and had significantly shorter operations (*p* = 0.005). Consultants performed the majority of operations from 2012 to 2019 (*p* < 0.001); however, no significant differences in patient gender, BMI, laterality, stone size or stone location were identified. No significant difference in mGy (*p* = 0.967), DAP (*p* = 0.164) or FT (*p* = 0.748) was identified for each time period, and similarly, no significant difference in mGy (*p* = 0.327), DAP (*p* = 0.479) or FT (*p* = 0.729) comparing specialty registrars only (Table 3).

**TABLE 2:** Baseline characteristics comparison between time periods with specialty registrar (urology 2020–2022 and general surgery 2012–2019).

	2020–2022	2012–2019	*p*‐value
Patients	126	284	
Age, years, median (IQR)	61 (25.25)	54 (21.75)	0.001
Gender, male (%)	92 (73)	185 (65.1)	0.072
BMI, median (IQR)	29.75 (8.17)	30.05 (8.5)	0.437
Side, *n* (%)	66 (52.4)	152 (53.5)	0.831
Case duration, minutes, median (IQR)	39 (13)	42 (14)	0.005
Size of stone, mm, median (IQR)	6 (4)	7 (4)	0.114
Stone location			0.309
Proximal, *n* (%)	66 (52.4)	144 (50.7)	
Mid, *n* (%)	42 (33.3)	106 (37.3)	
Distal, *n* (%)	18 (14.3)	34 (12)	
Consultant operator	67 (53.2)	181 (63.7)	<0.001

Abbreviation: IQR, interquartile range.

**TABLE 3:** Subgroup comparison of radiation exposure.

Operator level				
	Registrar	Consultant	*p*‐value	
*n*	162	248		
mGy, median (IQR)	11.8 (15.2)	13.1 (12.85)	0.671	
Gy/cm^2^, median (IQR)	2.76 (4.04)	3.15 (3.19)	0.613	
FT, median (IQR)	47.5 (43)	40.5 (44.25)	0.266	
Stone location				
	Proximal	Mid	Distal	*p*‐value
*n*	210	52	128	
mGy, median (IQR)	12.45 (12.83)	15.3 (14.895)	12.4 (14.7275)	0.997
Gy/cm^2^, median (IQR)	2.815 (3.01)	3.34 (4.225)	3.035 (3.4375)	0.743
FT, median (IQR)	39.5 (39.75)	48 (48.5)	50 (41.5)	0.892
Gender				
	Female	Male	*p*‐value	
*n*	133	277		
mGy, median (IQR)	12.3 (14.75)	12.6 (13.85)	0.438	
Gy/cm^2^, median (IQR)	2.995 (3.6475)	2.865 (3.28)	0.751	
FT, median (IQR)	49 (45)	40 (41)	0.527	
Laterality				
	Left	Right	*p*‐value	
*n*	218	192		
mGy, median (IQR)	11.9 (13.23)	13.6 (7.75)	0.995	
Gy/cm^2^, median (IQR)	2.72 (3.1)	3.24 (2.355)	0.843	
FT, median (IQR)	39 (41.5)	50 (28)	0.921	
2010–2019 vs. 2020–2022				
	2010–2019	2020–2022	*p*‐value	
*n*	284	126		
mGy, median (IQR)	12.6 (13.48)	12.5 (15.36)	0.967	
Gy/cm^2^, median (IQR)	2.78 (3.28)	3.285 (3.6)	0.164	
FT, median (IQR)	43.5 (43.25)	47 (47)	0.748	
General surgery registrar vs. urology registrar				
	General surgery registrar	Urology registrar	*p*‐value	
*n*	103	59		
mGy, median (IQR)	12.7 (14.545)	10.9 (14.41)	0.327	
Gy/cm^2^, median (IQR)	2.72 (4.28)	2.99 (3.22)	0.479	
FT, median (IQR)	47 (41.5)	48 (41)	0.729	
mGy				
	Pearson correlation coefficient	*p*‐value	Correlation strength	
Case duration	0.289	<0.001	Low	
BMI	0.290	<0.001	Low	
Stone size	0.023	0.639	Very low	
Age	0.020	0.020	Very low	
Gy/cm^2^ (DAP)				
	Pearson correlation coefficient	*p*‐value	Correlation strength	
Case duration	0.289	<0.001	Low	
BMI	0.287	<0.001	Low	
Stone size	0.18	0.723	Very low	
Age	0.14	0.773	Very low	
Fluoroscopy time				
	Pearson correlation coefficient	*p*‐value	Correlation strength	
Case duration	0.283	<0.001	Low	
BMI	−0.83	0.15	Very low	
Stone size	0.024	0.63	Very low	
Age	−0.019	0.697	Very low	

Abbreviations: DAP, dose area product; IQR, interquartile range.

### 3.3 Stone size

A median stone size of 7 mm was identified across all subgroups except for 2020–2022, which was 6 mm but was not significantly different (*p* = 0.114). Male gender was associated with a higher median stone size of 7 mm (IQR 4) versus 6 mm (IQR 4) (*p* = 0.001). No significant correlation was identified for increased age (*r* = 0.049 and *p* = 0.320) or increased BMI (*r* = 0.048 and *p* = 0.400). Stone size was largest in the proximal ureter 7 mm (IQR 4) compared to the mid ureter 6 mm (IQR 2) and distal ureter 6 mm (IQR 2), but did not reach statistical significance (*p* = 0.079). Laterality showed a larger stone size on the left median of 7 mm (IQR 10) compared with the right 6 mm (IQR 9) but similarly did not achieve statistical significance (*p* = 0.069).

### 3.4 Radiation exposure

Case duration had a significant correlation with increased mGy (*p* < 0.001), DAP (*p* < 0.001) and FT (<0.001), and similarly, so did BMI, but only for DAP (<0.001) and mGy (*p* < 0.001) but not FT (*p* = 0.15) (Table 3); however, the correlation strength was low for all *r* coefficients. Stone size had no significant correlation with mGy, DAP or FT and neither did age except for a negligible correlation with mGy (*r* = 0.020 and *p* = 0.020). No significant radiation exposure difference was identified for gender mGy, DAP or FT. Stone location in the proximal, mid or distal ureter was not significantly associated with increased mGy (*p* = 0.997), DAP (*p* = 0.743) or FT (*p* = 0.892). Laterality of stone similarly showed no significant association for increased mGy (*p* = 0.995), DAP (*p* = 0.843) or FT (*p* = 0.921), nor gender mGy (*p* = 0.438), DAP (*p* = 0.751) or FT (*p* = 0.527).

## 4 DISCUSSION

Radiation exposure in this study population of FT 44.5 s (IQR 44 s) and DAP 2.94 Gy/cm^2^ (IQR 3.48 Gy/cm^2^) met the UK multi‐centre FLASH study^9^ reference standards of FT 49 s and DAP 2.3. Furthermore, this study demonstrated no significant difference comparing consultant to registrar FT or DAP, in comparison to the FLASH study, which identified a significant difference of DAP 2.17 Gy/cm^2^ and FT 41 s for consultants and for registrars DAP 1.38 Gy/cm^2^ FT 26 s. Our findings had comparable consultant values, but not registrar values with higher DAP and FT for registrars. The FLASH study notes that this could be explained by the greater complexity of consultant cases compared to the registrar, which is also a limitation of this study.

Urolithiasis in Australia has an incidence of 131 per 100 000^11^ but has seen increasing use of retrograde intrarenal surgery (RIRS) to manage urolithiasis in Australia.^6^, ^12^ This is reflected internationally, as advancements in endo‐urological technology have led to guidelines adopting and advocating for minimally invasive surgery and RIRS.^13^ With the increased usage of RIRS subsequently the use of fluoroscopy has also increased, with the UK multi‐centre FLASH study^9^ developing reference standards to allow practitioners to self‐audit radiation exposure and change practise as necessary to mitigate risk.

Limited literature has examined radiation exposure to urologists, with the majority of studies in orthopaedic surgery, which has demonstrated a significant fivefold increased risk of cancer in orthopaedic surgeons.^14^ Seniority in orthopaedic surgery has also demonstrated significantly reduced fluoroscopy usage and radiation exposure with senior operator experience in emergency^15^ settings. However, few studies involve urologists during RIRS. A study by Park^8^ demonstrated that a surgeon can perform up to 517 RIRS with appropriate radiation protection; however, this amount drops by 85% without shielding. Predictors for increased radiation exposure have been examined for percutaneous nephrolithotomy (PCNL), which suggests risk factors being an increased stone burden, staghorn calculi and longer procedural time.^16^ However, the same study also compared registrar to consultant urologist and found no significant difference or significant risk factors when corrected for confounders with multivariate analysis. Similar to this study's findings, BMI and case duration were risk factors for increased radiation exposure^17^ in PCNL. A US study that audited urology resident radiation exposure over a 60‐day period determined that while they were within annual radiation thresholds, resident physicians exceed the exposure of consultants by up to 20–25 fold^18^ (Data S1). Possible reasons include the consultant urologist's knowledge, experience and familiarity with equipment, which can all affect radiation usage and exposure.^19^ Non‐technical skills can affect radiation use, as effective communication and teamwork with the radiographer are essential to efficiently coordinate fluoroscopy usage^20^ irrespective of the primary fluoroscopy controller, being the radiographer or surgeon.^21^ Suggestions to reduce radiation exposure include education and fluoroscopy protocols,^22^ and also formal feedback to surgeons, which has been demonstrated to reduce FT by 24%.^23^ Comparisons between urology and general surgery registrars have been examined in the context of the acute scrotum for emergency cross‐cover of surgical specialties^24^ but not for emergency stenting. Basic emergency urology covered by general surgery does occur in resource‐limited areas such as in the developing world, or in rural areas such as in this study. In this institution, registrars were supervised and trained in both elective and emergency settings to attain competency in emergency ureteric stenting prior to independence. Although this study identifies safety for general surgery registrars to perform ureteric stenting, a limitation is it did not examine or compare patient outcomes.

Radiation as a silent hazard is important for both surgeons^25^ and patients, especially when considering paediatric patients,^26^ younger age of urology registrars and pregnant endourologists^27^ who may require lower radiation exposure dose thresholds or exemptions. Furthermore, other members of the operating theatre are also exposed including nursing staff and anaesthetics. Compliance with radiation safety practises is essential for theatre environments with accreditation maintained by Ionising Radiation Safety Committees through radiation safety officers and compliance audits. However, amongst healthcare professionals literature has determined that both knowledge and compliance are substandard with 44.1% lacking formal training in a UK survey^28^ including urology consultants, registrars, nurses, anaesthetics and radiographers. Furthermore, consultants were more likely to use dosimeters compared with registrars with up to 70% reporting never used one.^29^ A similar survey in the United States demonstrated^30^ insufficient knowledge with only 13% undergoing formal radiation safety training and only 54% answering questions correctly about radiation travel and exposure. However, in spite of this, compliance with lead aprons and thyroid shields is high,^29^ up to 99% and 73% respectively, despite up to 56% never being issued a dosimeter.

Stone characteristics as risk factors for increased radiation during RIRS and lithotripsy have been examined in the literature, with greater radiation exposure associated with renal stones compared to ureteral stones and stone diameter >10 mm.^31^ These findings are not reflected this our study, likely due to a median stone size of less than 10 mm and the exclusion of primary lithotripsy in the study population.

A limitation of this study was that personal dosimeters were not utilised as this was a retrospective audit, and individual dosimeters could not be allocated and recorded. Concern for confounding exists as consultant urologists operated on patients with greater BMI, and consultants would likely tend to operate on more complex cases or take over mid‐case if difficulties arose, a limitation also addressed in the FLASH study. A multivariate analysis to correct for confounders was performed; however, other unrecorded confounders exist that can increase case difficulty, including anatomical variation such as a narrow tortuous ureter or orifice. Furthermore, distal ureteric stones made up a greater proportion of the total caseload for registrars compared with consultants, likely due to primary removal via ureteroscopy being performed by consultants, and these cases were excluded as primary ureteroscopy was an exclusion criterion for this study. Ureteroscopy is a major urological procedure with a higher risk than stenting; however with proficiency has been demonstrated in the literature to be a safe and feasible operation with appropriate patient selection.^32^

An assessment of true radiation exposure to surgeons could not be performed because of retrospective data collection and no formal allocation of dosimeters. An informal assessment utilising the study by Park^8^ of a 98% lead apron and 97% thyroid shield radiation reduction demonstrates that with a mean of 33 stents per year and median mGy exposure of 11.8 mGy, an annual 12.474 mGy exposure can be calculated from all stents. While this value is compliant with the ARPANSA annual threshold, this is still high considering this calculation only provides an incomplete assessment of true radiation exposure without factors such as distance from the C‐arm or compliance with lead shielding. Furthermore, it does not account for elective fluoroscopy usage and thus a total assessment of annual radiation cannot be completed. Thus, we advocate for personal dosimeter usage for all surgeons, consultants and registrars, to prospectively assess true radiation exposure.

## 5 CONCLUSION

Radiation exposure during emergency ureteric stenting for urolithiasis demonstrates no significant difference between a consultant or registrar under a urologist's supervision. Despite this, we suggest formal radiation safety education for all health professionals involved with intra‐operative fluoroscopy and the appropriate use of personal dosimeters.

## AUTHOR CONTRIBUTIONS

**Kevin Tree:** Conceptualisation; methodology; investigation; data curation; formal analysis; writing—original draft; writing—review and editing. **Nicholas Chang:** Methodology; investigation; data curation; writing—review and editing. **Roy Huynh:** Methodology; investigation; data curation; formal analysis; writing—review and editing. **Balasubramaniam Indrajit:** Conceptualisation; methodology; writing—review and editing; supervision. **Dean Fisher:** Conceptualisation; methodology; writing—review and editing; supervision. **Sris Baskaranathan:** Conceptualisation; methodology; writing—review and editing; supervision.

## CONFLICT OF INTEREST STATEMENT

The authors declare no conflicts of interest in preparing this article.

## Supporting information


**Data S1.** Supporting Information
