# Supplementary material for: Radiation exposure in emergency ureteric stenting: A subgroup analysis by operator
Source: BJUI Compass. 2023 Apr 29;4(6):680–7. doi: 10.1002/bco2.245 (PMC10560617; doi:10.1002/bco2.245)
Supplement: Supplementary file 1 — Data S1. Supporting Information [file BCO2-4-680-s001.docx]

Supplementary Material

**S1 Nomenclature Clarification**

In the Australian healthcare system, the role of an “accredited registrar” is a doctor that is formally accredited into a specialty training program as a trainee, and is comparable to a resident in the United States medical system.

An “unaccredited registrar” are doctors who fulfil greater duties and responsibilities similar to an “accredited registrar” but are not formally enrolled into a specialty training program.
